# Unmet Needs for Ancillary Services by Provider Type Among People With Diagnosed Human Immunodeficiency Virus

**DOI:** 10.1093/ofid/ofae284

**Published:** 2024-05-14

**Authors:** Celina Thomas, Xin Yuan, Jennifer A Taussig, Yunfeng Tie, Sharoda Dasgupta, David J Riedel, John Weiser

**Affiliations:** Department of Medicine, University of Maryland School of Medicine, Baltimore, Maryland, USA; DLH Corporation, Atlanta, Georgia, USA; Division of HIV Prevention, Centers for Disease Control and Prevention, Atlanta, Georgia, USA; Division of HIV Prevention, Centers for Disease Control and Prevention, Atlanta, Georgia, USA; Division of HIV Prevention, Centers for Disease Control and Prevention, Atlanta, Georgia, USA; Department of Medicine, University of Maryland School of Medicine, Baltimore, Maryland, USA; Institute of Human Virology, University of Maryland School of Medicine, Baltimore, Maryland, USA; Division of HIV Prevention, Centers for Disease Control and Prevention, Atlanta, Georgia, USA

**Keywords:** ancillary services, HIV/AIDS, nurse practitioners, physician assistants, unmet needs

## Abstract

**Background:**

Unmet needs for ancillary services are substantial among people with human immunodeficiency virus (PWH), and provider type could influence the prevalence of unmet needs for these services.

**Methods:**

Data from a national probability sample of PWH were analyzed from the Centers for Disease Control and Prevention’s Medical Monitoring Project. We analyzed 2019 data on people who had ≥1 encounter with a human immunodeficiency virus (HIV) care provider (N = 3413) and their care facilities. We assessed the proportion of needs that were unmet for individual ancillary services, overall and by HIV care provider type, including infectious disease (ID) physicians, non-ID physicians, nurse practitioners, and physician assistants. We calculated prevalence differences (PDs) with predicted marginal means to assess differences between groups.

**Results:**

An estimated 98.2% of patients reported ≥1 need for an ancillary service, and of those 46% had ≥1 unmet need. Compared with patients of ID physicians, needs for many ancillary services were higher among patients of other provider types. However, even after adjustment, patients of non-ID physicians had lower unmet needs for dental care (adjusted PD, −5.6 [95% confidence interval {CI}, −9.9 to −1.3]), and patients of nurse practitioners had lower unmet needs for HIV case management services (adjusted PD, −5.4 [95% CI, −9.4 to −1.4]), compared with patients of ID physicians.

**Conclusions:**

Although needs were greater among patients of providers other than ID physicians, many of these needs may be met by existing support systems at HIV care facilities. However, additional resources may be needed to address unmet needs for dental care and HIV case management among patients of ID physicians.

## Teaser

Unmet needs for most ancillary services were comparable among all provider types. However, additional resources may be needed to address remaining disparities in unmet needs for dental care and HIV case management among patients of infectious diseases physicians.

Ancillary services, including human immunodeficiency virus (HIV) case management, mental health services, and housing and food assistance, support retention in HIV medical care, adherence to antiretroviral therapy (ART), and viral suppression [1], and advance the national goal of improving HIV-related outcomes for people with HIV (PWH). An estimated 48% of PWH report at least 1 unmet need for ancillary services [2]. Certain populations of PWH, such as those who identify as Black/African American, people experiencing homelessness, those living in a household at or below the poverty threshold, and people who inject drugs, were more likely to report unmet needs [1]. These same groups of PWH are less likely to have sustained viral suppression [3–5].

The Ryan White HIV/AIDS Program (RWHAP) provides funding for clinical and ancillary services for PWH in the United States (US) who are uninsured and underinsured [6–9]. Uninsured PWH who receive RWHAP assistance have fewer unmet needs for HIV clinical support services and other medical services than PWH who are uninsured and not receiving RWHAP assistance [6]. The type of HIV care provider might also influence the burden of unmet needs for ancillary services, but this has not been assessed. Previous studies examining associations between provider characteristics and HIV outcomes have shown that patients of providers with more experience treating HIV have better outcomes, including higher levels of viral suppression, lower costs, and prolonged survival [10–13]. Healthcare providers are responsible for initiating treatment plans that address patients’ needs, including ordering diagnostic/screening tests, prescribing medications, performing procedures, and when necessary, referring for services that providers cannot offer themselves. Providers typically first identify patient needs by taking a thorough social history and assessing barriers to care. Once a need is identified, providers should identify resources available to address the need, refer the patient, and communicate to the patient the importance of addressing the need. Meeting PWH where they are to address barriers to care related to social determinants of health (SDOH) requires a systems-level approach that can begin with providers [10–13].

In this analysis, we examined total needs and unmet needs for ancillary services by provider type, among a representative sample of US adults with HIV.

## METHODS

### Design and Data Collection

The Centers for Disease Control and Prevention’s (CDC) Medical Monitoring Project (MMP) is an annual, cross-sectional survey designed to produce nationally representative estimates of the behavioral and clinical characteristics of adults with diagnosed HIV in the US. MMP data collection constitutes routine public health surveillance and was thus determined by the CDC to be nonresearch. This activity is conducted consistent with the applicable federal law and CDC policy [14]. When required, participating jurisdictions obtained local institutional review board approval to collect data. All participants provided informed consent.

MMP methods have been previously described [15]. In brief, MMP uses 2-stage sampling in which, during the first stage, 16 states—including 6 separately funded metropolitan areas within selected states—and 1 territory were sampled from all states, the District of Columbia, and Puerto Rico. During the second stage, simple random samples of people with diagnosed HIV aged ≥18 years (PWH) were drawn annually for each participating area from the National HIV Surveillance System, a census of people with diagnosed HIV in the US. People sampled during the 2019 data collection cycle were recruited for a phone or face-to-face interview. Medical records were then abstracted at the facility identified by participants as their most frequent source of HIV care in the previous 2 years. Data were collected during June 2019–May 2020. Medical record data included all outpatient encounters with medical providers, including the provider's profession, during a 2-year, retrospective observation period ending on the interview date (end of observation period).

All sampled areas and separately funded jurisdictions within states participated in MMP, including California (including Los Angeles County and San Francisco), Delaware, Florida, Georgia, Illinois (including Chicago), Indiana, Michigan, Mississippi, New Jersey, New York (including New York City), North Carolina, Oregon, Pennsylvania (including Philadelphia), Puerto Rico, Texas (including Houston), Virginia, and Washington. The first-stage response rate was 100%. In all, 4100 sampled people were interviewed, for a second-stage participant response rate of 45%.

In a supplemental MMP facility survey conducted in 2021, we surveyed representatives at the 1023 medical care facilities identified by MMP participants from the 2019 data cycle as their most frequent source of HIV care in the previous 2 years. Data were collected on characteristics of these HIV care facilities. In brief, data were collected online or via mail or phone and assessed for the availability of clinical and supportive services onsite and through established referrals. The facility survey response rate was 45%. To generate patient-level estimates for all participants, we imputed missing data using 2 steps: recursive partitioning (trees) to create imputation classes and weighted sequential hot deck to produce the imputed values. This ensured that patient-level facility estimates had no missing values and reduced nonresponse bias. MMP facility survey methods have been previously described in detail [16].

### Measures

Variables related to demographic characteristics and SDOH included age, gender (male, female, or transgender), race/ethnicity (non-Hispanic White; non-Hispanic Black/African American, Hispanic/Latino of any race, American Indian/Alaska Native, Asian, Native Hawaiian/other Pacific Islander, and multiracial); household income (at or below the federal poverty level [FPL] or above the FPL); educational attainment (less than high school, high school diploma or General Educational Development, or more than high school); health insurance or coverage type (any private, public only excluding RWHAP assistance [Medicaid, Medicare, Tricare/Civilian Health and Medical Program of the Uniformed Services and/or Veterans Administration, and other or unknown public insurance], RWHAP/AIDS Drug Assistance Program [ADAP] only, or no insurance or coverage); homelessness; and incarceration. All demographic characteristics and SDOH were based on the past 12 months.

Clinical variables obtained by self-report included binge drinking in the past 30 days, any drug use in the past 12 months, any injection drug use in the past 12 months, symptoms of major or other depression in the past 2 weeks based on the Patient Health Questionnaire-8 scale [17], and symptoms of moderate or severe anxiety in the past 2 weeks based on the Generalized Anxiety Disorder-7 scale [18]. We also ascertained whether the facility where patients reported receiving most of their HIV care in the previous 2 years received any RWHAP funding.

Need for and receipt of ancillary services was assessed through self-report based on the past 12 months. For each service type, participants were asked if they received the service; persons who did not receive the service were asked if they needed the service. Total (any) need was defined as needing a service, regardless of whether it was received. Among those with any need, unmet need was defined as needing but not receiving a particular service. Ancillary services were categorized into 3 domains: HIV clinical support services (medicine through ADAP, HIV case management, adherence support services, HIV peer support, and patient navigation services); other medical services (dental care, mental health services, and drug or alcohol counseling or treatment); and subsistence services (Supplemental Nutrition Assistance Program or the Special Supplemental Nutrition Program for Women, Infants, and Children, transportation assistance, meal or food services, and shelter or housing services).

Clinicians with prescribing privileges were classified as HIV care providers if they were identified as such by HIV care facility leadership. Medical record documentation of ordering a CD4^+^ lymphocyte cell (CD4) count or HIV RNA test or prescribing ART did not in itself constitute being an HIV care provider. We categorized PWH who had ≥1 encounter with an HIV care provider of known profession during the past 12 months by type of provider(s) performing the encounters: ID physician only, non-ID physician only, nurse practitioner (NP) only, physician assistant (PA) only, or a combination of ID physician and NP and/or PA (mixed providers). Patients of other combinations of provider types were excluded because of small sample sizes.

### Statistical Analysis

Data of MMP participants were weighted based on known probabilities of selection at the state/territory and person levels, adjusted for nonresponse, and poststratified to known population totals by age, race/ethnicity, and sex at birth from the National HIV Surveillance System (NHSS). This design allows for reporting of representative estimates among all adults with diagnosed HIV in the US [2, 19].

Among 4100 adults with diagnosed HIV interviewed for the 2019 MMP data cycle, we assessed the prevalence of ≥1 unmet need within categories of ancillary services, including HIV support services, non-HIV medical services, and subsistence services, overall and by demographic and clinical characteristics and SDOH. Among 3413 persons who had ≥1 encounter with an HIV care provider of known type during the past 12 months, we calculated the percentages with needs for individual ancillary services and, among those with needs, the percentages whose needs were unmet, overall and by provider type. We calculated weighted percentages with 95% confidence intervals (CIs) for all characteristics and used logistic regression with predicted marginals to calculate prevalence differences (PDs) with 95% CIs to quantify absolute differences between groups. All models assessing needs by provider type presented PDs using ID physicians as the referent group. We reported unadjusted estimates of the burden of total needs (ie, regardless of whether needs are met or unmet). To identify factors independently associated with unmet needs, we adjusted for whether facilities where patients received HIV care provided the corresponding ancillary service onsite and received RWHAP funding in the past 2 years, and for SDOH and selected demographic and clinical characteristics, including race/ethnicity, age, income above or below the FPL, incarceration, homelessness, drug use in the past 12 months, and depression symptoms in the past 2 weeks, that were associated with having unmet needs in the current analysis and with provider type in a previous analysis [20]. Models assessing differences in total needs (ie, regardless of whether needs are met or unmet) by provider type were not adjusted. These data were used to assess total burden of needs by provider type, rather than to investigate if provider type is independently associated with total needs. We considered PDs ≥5 percentage points with 95% CIs not containing the null to be meaningful from a public health perspective. All analyses were conducted using SAS survey procedures and SAS-callable SUDAAN.

## RESULTS

Among all PWH, 98.2% (95% CI, 97.6%–98.8%) had ≥1 need for ancillary services. Among those, 45.9% (95% CI, 41.8%–49.9%) reported at least 1 unmet need for ancillary services (not displayed in tables). The prevalence of having ≥1 unmet need varied across many characteristics and outcomes as shown in Supplementary Table 1. Notably, unmet needs for clinical support services, other medical services, and subsistence services were higher among people who recently experienced homelessness, those who used drugs for nonmedical purposes—including those who injected drugs—and persons who experienced symptoms of depression or anxiety, compared with other persons. Unmet needs for clinical support services and subsistence services were higher among persons living in households at or below the FPL and those who were recently incarcerated, compared with other persons.

Overall, dental care was the service with the highest reported total need (82.1%; Table 1). There was also substantial need for HIV case management services (65.1%), medicine through ADAP (52.9%), mental health services (40.6%), and medication adherence support services (37.2%). Additionally, transportation assistance was needed by 31.9%, meal or food services by 28.3%, shelter or housing services by 25.7%, HIV peer group support by 19.3%, and patient navigation services by 19.6%. Drug or alcohol counseling or treatment (8.7%) was the least reported need.

**Table 1.** Total Needs^a^ for Ancillary Services Among Adults With Diagnosed Human Immunodeficiency Virus (HIV) Who Had ≥1 Encounter With an HIV Care Provider of Known Type in the Past 12 Months—Medical Monitoring Project, United States, 2019 (N = 3413)

Total (n = 3413)^b^		ID Physician Only (n = 1528)		Other Physician Only (n = 576)				NP Only (n = 478)				PA Only (n = 124)				ID Physician and Either NP or PA (n = 336)			
No.	Col. % (95% CI)^c^	No.	Col. % (95% CI)	No.	Col. % (95% CI)	PD (vs ID) (95% CI)	*P* Value	No.	Col. % (95% CI)	PD (vs ID) (95% CI)	*P* Value	No.	Col. % (95% CI)	PD (vs ID) (95% CI)	*P* Value	No.	Col. % (95% CI)	PD (vs ID) (95% CI)	*P* Value
HIV case management services																			
2232	65.1 (61.3–68.9)	925	59.9 (55.1–64.7)	377	68.3 (55.7–81.0)	8.5 (−5.1 to 22.1)	.2229	343	70.6 (63.9–77.4)	10.8 (4.3–17.2)	.0011	70	58.1 (47.7–68.6)	−1.8 (−12.5 to 9.0)	.7496	243	71.1 (64.9–77.4)	11.2 (3.9–18.6)	.0028
Medicine through ADAP																			
1785	52.9 (50.4–55.3)	725	46.4 (42.9–49.8)	317	58.8 (51.1–66.5)	12.5 (2.7–22.2)	.0123	274	58.6 (51.3–65.8)	12.2 (4.4–20.0)	.0021	70	62.2 (53.4–71.0)	15.9 (6.1–25.6)	.0014	197	59.0 (53.2–64.7)	12.6 (7.1–18.1)	<.0001
Professional help remembering to take HIV medicines or correctly (adherence support services)																			
1261	37.2 (34.1–40.3)	489	32.3 (29.5–35.2)	244	43.6 (30.3–56.8)	11.3 (−2.9 to 25.4)	.1183	198	42.9 (38.8–46.9)	10.5 (5.5–15.6)	<.0001	42	34.6 (24.4–44.7)	2.2 (−9.5 to 14.0)	.7095	130	39.0 (32.9–45.0)	6.7 (.3–13.1)	.0418
HIV peer group support																			
662	19.3 (17.4–21.1)	267	18.0 (15.8–20.1)	124	21.1 (15.6–26.6)	3.1 (−3.1 to 9.3)	.3243	99	19.9 (14.1–25.7)	1.9 (−3.4 to 7.2)	.4805	18	14.2 (9.2–19.1)	−3.8 (−9.2 to 1.7)	.1728	78	23.2 (17.3–29.0)	5.2 (−1.7 to 12.1)	.14
Patient navigation services																			
684	19.6 (17.2–22.1)	280	17.7 (14.7–20.7)	111	19.3 (13.4–25.1)	1.6 (−5.7 to 8.8)	.6734	110	22.8 (17.0–28.7)	5.1 (.1–10.1)	.0477	20	17.4 (11.9–22.9)	−0.3 (−6.9 to 6.2)	.9202	72	21.2 (15.5–26.9)	3.5 (−1.8 to 8.8)	.1976
Dental care																			
2825	82.1 (79.5–84.7)	1261	82.5 (80.1–85.0)	501	86.6 (83.1–90.1)	4.0 (.4–7.7)	.03	379	76.3 (70.4–82.2)	−6.3 (−12.1 to –.4)	.035	97	75.1 (65.4–84.8)	−7.4 (−16.9 to 2.1)	.1261	275	82.6 (78.2–87.0)	0.1 (−5.0 to 5.1)	.9803
Mental health services																			
1410	40.6 (36.5–44.6)	574	36.7 (33.4–39.9)	291	50.3 (38.6–62.1)	13.7 (1.6–25.7)	.0264	183	39.1 (31.7–46.6)	2.5 (−4.0 to 9.0)	.4573	39	32.5 (22.0–43.0)	−4.2 (−13.2 to 4.8)	.3648	153	45.5 (39.9–51.1)	8.8 (2.5–15.2)	.0061
Drug or alcohol counseling or treatment																			
308	8.7 (7.2–10.1)	123	7.4 (5.9–8.9)	71	12.3 (8.3–16.3)	4.9 (1.0–8.8)	.0143	38	7.9 (5.1–10.8)	0.6 (−2.6 to 3.7)	.7273	8	7.5 (3.9–11.0)	0.1 (−3.9 to 4.0)	.9748	32	9.9 (6.1–13.7)	2.5 (−1.3 to 6.3)	.2031
Transportation assistance																			
1106	31.9 (29.8–34.1)	469	30.8 (27.7–33.8)	171	28.3 (23.8–32.7)	−2.5 (−7.8 to 2.8)	.3574	160	33.1 (27.4–38.8)	2.3 (−4.2 to 8.8)	.487	36	28.5 (20.5–36.6)	−2.2 (−11.9 to 7.4)	.6516	120	34.6 (29.4–39.9)	3.9 (−2.2 to 9.9)	.2091
Meal or food services																			
986	28.3 (25.8–30.8)	434	27.8 (25.3–30.4)	136	24.0 (19.5–28.6)	−3.8 (−8.6 to 1.0)	.1193	149	31.6 (25.0–38.3)	3.8 (−2.1 to 9.7)	.2053	33	26.2 (16.4–36.1)	−1.6 (−11.3 to 8.2)	.7504	103	29.1 (24.2–34.0)	1.3 (−4.0 to 6.6)	.6355
Shelter or housing services																			
904	25.7 (23.1–28.3)	412	25.8 (22.9–28.7)	124	22.0 (16.1–27.9)	−3.8 (−10.1 to 2.4)	.2303	133	25.7 (20.6–30.9)	−0.1 (−4.4 to 4.3)	.9712	31	24.8 (15.3–34.4)	−1.0 (−11.6 to 9.6)	.8535	89	25.9 (21.2–30.6)	0.1 (−5.4 to 5.6)	.9698

Abbreviations: ADAP, AIDS Drug Assistance Program; CI, confidence interval; Col. %, column percentage; HIV, human immunodeficiency virus; ID, infectious diseases; No., sample size; NP, nurse practitioner; PA, physician assistant; PD, prevalence difference.

^a^Needs for services during the past 12 months were assessed on a self-report basis. Total need was defined as needing a service, regardless of whether it was received.

^b^All n values are unweighted. The combined number of patients of various provider types does not equal the total of all patients because 371 patients (10.9%) with other combinations of provider types were excluded.

^c^All percentages are weighted.

The services with the highest percentage of needs that were unmet were HIV peer group support at 34.1% and shelter and housing services at 31.1% (Table 2). Other services with a substantial percentage of unmet needs were meal or food services (25.7%), dental care (25.0%), transportation services (20.8%), mental health services (19.8%), patient navigation services (18.5%), and drug or alcohol counseling or treatment (17.5%). The lowest percentages of unmet needs were for HIV case management services (8.0%), medicine through ADAP (3.8%), and adherence support services (1.4%).

**Table 2.** Adjusted Total of Needs for Ancillary Services That Were Unmet^a^ Among People With Human Immunodeficiency Virus (HIV) Who Had ≥1 Encounter With an HIV Care Provider of Known Type in the Past 12 Months—Medical Monitoring Project, United States, 2019 (N = 3413)

Total (n = 3413)^b^		ID Physician Only (n = 1528)		Other Physician Only (n = 576)				NP Only (n = 478)				PA Only (n = 124)				ID Physician and Either NP or PA (n = 336)			
No.	Col. % (95% CI)^c^	No.	Col. % (95% CI)	No.	Col. % (95% CI)	aPD (vs ID) (95% CI)	*P* Value	No.	Col. % (95% CI)	aPD (vs ID) (95% CI)	*P* Value	No.	Col. % (95% CI)	aPD (vs ID) (95% CI)	*P* Value	No.	Col. % (95% CI)	aPD (vs ID) (95% CI)	*P* Value
HIV case management services																			
171	8.0 (5.7–10.2)	86	10.2 (7.0–13.3)	21	5.5 (1.6–9.4)^d^	−3.2 (−7.6 to 1.1)	.1417	17	4.4 (2.3–6.5)	−5.4 (−9.4 to −1.4)	.0086	5	7.5 (.7–14.3)^d^	−3.2 (−10.4 to 4.1)	.3929	22	10.1 (5.7–14.6)	0.2 (−4.2 to 4.7)	.9204
Medicine through ADAP																			
66	3.8 (3.0–4.6)	32	4.6 (3.0–6.2)	13	4.9 (1.6–8.2)^d^	0.1 (−7.4 to 7.5)	.9868	9	2.6 (1.4–3.8)	−0.7 (−8.1 to 6.6)	.8457	1	1.0 (.0–3.0)^d^	−2.9 (−14.4 to 8.6)	.6196	5	2.8 (.0–5.7)^d^	−2.1 (−10.0 to 5.7)	.5902
Professional help remembering to take HIV medicines or correctly (adherence support services)																			
16	1.4 (.7–2.1)	10	2.0 (.7–3.4)^d^	1	0.9 (.0–2.1)^d^	−0.8 (−10.8 to 9.3)	.8835	2	0.8 (.0–1.8)^d^	−1.2 (−21.1 to 18.7)	.9044	0	…	…	…	0	…	…	…
HIV peer group support																			
211	34.1 (28.8–39.3)	84	34.5 (27.7–41.4)	44	36.9 (27.4–46.4)	2.5 (−7.8 to 12.7)	.6373	32	33.7 (22.8–44.6)	−1.4 (−14.1 to 11.4)	.8345	3	14.9 (1.5–28.3)^d^	−20.4 (−39.2 to −1.6)	.034	26	36.0 (24.2–47.8)	5.0 (−8.4 to 18.4)	.4658
Patient navigation services																			
122	18.5 (13.1–23.8)	51	19.4 (11.4–27.3)	20	18.1 (8.8–27.3)	0.5 (−8.0 to 8.9)	.9135	20	17.5 (10.2–24.7)	−0.4 (−10.9 to 10.1)	.9399	2	8.0 (.0–18.5)^d^	−5.4 (−24.4 to 13.7)	.5822	14	18.3 (8.8–27.7)	2.4 (−8.7 to 13.6)	.6681
Dental care																			
668	25.0 (22.3–27.8)	306	25.5 (22.5–28.6)	95	19.0 (15.4–22.7)	−5.6 (−9.9 to −1.3)	.0114	112	30.4 (25.0–35.9)	3.2 (−2.8 to 9.1)	.2954	27	27.5 (15.8–39.2)	0.8 (−12.6 to 14.2)	.9087	67	25.8 (20.2–31.4)	−2.4 (−8.0 to 3.2)	.4024
Mental health services																			
269	19.8 (15.7–23.9)	111	20.8 (16.0–25.6)	59	18.2 (7.3–29.0)^d^	2.2 (−6.1 to 10.5)	.6078	42	22.4 (16.5–28.2)	2.8 (−5.3 to 10.9)	.4968	12	29.7 (15.2–44.2)	8.8 (−3.3 to 20.9)	.1535	29	21.2 (12.7–29.8)	3.0 (−4.6 to 10.6)	.4383
Drug or alcohol counseling or treatment																			
52	17.5 (11.4–23.5)	19	17.0 (8.8–25.3)	16	21.1 (4.0–38.2)^d^	−0.4 (−17.4 to 16.5)	.9588	7	18.6 (5.1–32.1)^d^	1.8 (−14.5 to 18.2)	.8285	2	32.1 (.0–64.6)^d^	9.8 (−26.1 to 45.8)	.5918	4	9.2 (.2–18.2)^d^	−6.1 (−25.6 to 13.4)	.5388
Transportation assistance																			
217	20.8 (18.4–23.2)	86	20.0 (16.1–23.9)	39	23.0 (14.9–31.1)	4.5 (−7.1 to 16.1)	.4481	32	20.6 (14.4–26.9)	−1.0 (−8.9 to 7.0)	.8155	8	23.6 (11.0–36.2)	2.6 (−8.2 to 13.4)	.6319	32	25.3 (16.6–34.1)	8.1 (−1.8 to 17.9)	.1089
Meal or food services																			
239	25.7 (22.1–29.2)	99	24.4 (18.3–30.5)	37	24.7 (18.0–31.4)	1.9 (−9.4 to 13.2)	.739	44	31.7 (24.0–39.3)	7.2 (−1.5 to 16.0)	.1063	9	33.6 (16.0–51.2)^d^	6.7 (−14.2 to 27.7)	.5289	30	28.0 (18.6–37.4)	2.6 (−9.7 to 14.9)	.6798
Shelter or housing services																			
274	31.1 (25.9–36.2)	127	32.1 (25.1–39.0)	29	23.1 (14.7–31.4)	−6.6 (−17.5 to 4.2)	.2311	45	33.6 (23.5–43.7)	−1.4 (−9.8 to 7.0)	.7446	12	36.8 (19.0–54.5)^d^	−5.0 (−20.5 to 10.6)	.5339	33	39.5 (28.5–50.5)	8.6 (−3.5 to 20.6)	.1643

Data were adjusted for covariates included in models: age; race/ethnicity; income above or below the federal poverty threshold; incarceration, homelessness, or drug use in the past 12 months; depression symptoms in the past 2 weeks; and whether the facility where the person received most of their HIV care in the past 2 years received any Ryan White HIV/AIDS Program funding and provided the particular ancillary service onsite.

Abbreviations: ADAP, AIDS Drug Assistance Program; aPD, prevalence difference; CI, confidence interval; Col. %, column percentage; HIV, human immunodeficiency virus; ID, infectious diseases; No., sample size; NP, nurse practitioner; PA, physician assistant.

^a^An unmet need was characterized by needing a service, but not receiving that service, and was calculated among persons with any need for a particular service.

^b^All n values are unweighted.

^c^All percentages are weighted.

^d^Coefficient of variation is ≥0.3, absolute CI width is ≥0.30, or absolute CI width is between 0.05 and 0.30 and relative CI width is >130%. Estimate should be interpreted with caution.

Total need for certain ancillary services differed based on provider type. For example, compared with patients of ID physicians, patients of non-ID physicians, NPs, PAs, and the mixed provider group (ID physician + NP/PA) were more likely to need medicine through ADAP (PD range, 12.2–15.9). Patients of non-ID physicians also had more need for mental health services (PD, 13.7 [95% CI, 1.6–25.7]). Patients of NPs had more need for HIV case management (PD, 10.8 [95% CI, 4.3–17.2]), adherence support (PD, 10.5 [95% CI, 5.5–15.6]), and patient navigation (PD, 5.1 [95% CI, .1–10.1]). Patients in the mixed provider group demonstrated more need for HIV case management (PD, 11.2 [95% CI, 3.9–18.6]), adherence support (PD, 6.7 [95% CI, .3–13.1]), and mental health services (PD, 8.8 [95% CI, 2.5–15.2]).

Unmet needs models were adjusted for differences in patient characteristics by provider type, including race/ethnicity, income above or below the FPL, age, incarceration, homelessness, drug use in the past 12 months, and depression symptoms in the past 2 weeks. In addition, models were adjusted for whether the facility where the person received most of their HIV care in the past 2 years received any RWHAP funding and provided the corresponding needed ancillary service onsite. Even after adjustment, compared with patients of ID physicians, patients of non-ID physicians who needed dental care were less likely for the need to be unmet (adjusted PD, −5.6 [95% CI, −9.9 to −1.3), and patients of NPs who needed HIV case management services were less likely for that need to be unmet (aPD, −5.4 [95% CI, −9.4 to −1.4]).

## DISCUSSION

This study has several important findings. Compared with patients of ID physicians, patients of other provider types were more likely to need 1 or more ancillary services. Encouragingly, despite these higher levels of needs, the data showed no differences in unmet needs for many ancillary services by provider type. Our results show there may be disparities for unmet needs for HIV case management and dental care by provider type. After accounting for differences in demographic and clinical characteristics, SDOH, health insurance type, facility RWHAP funding status, and the availability of specific ancillary services onsite at the facility where patents received their HIV care, patients of NPs who needed HIV case management were less likely than patients of ID physicians to have that need unmet. Similarly, patients of non-ID physicians who needed dental care were less likely to have that need unmet. Since ancillary services are a crucial component of HIV care, increasing the ability of all providers to meet the service needs of their patients may increase engagement in care, adherence to medications, viral suppression, and quality of life for PWH.

The need for ancillary services was particularly high among patients of non-ID physicians, NPs, PAs, and the mixed provider group. This is likely due to differences in SDOH among patients of different provider types, as we did not adjust for SDOH when calculating total needs for services. Patients with social determinants of poor health are more likely to have service needs and are more likely to receive care at RWHAP-funded facilities [8], where the percentage of non-ID physicians, NPs, and PAs is higher [21].

Our findings add to the current body of literature on differences in care provision by provider type. Previous studies have shown that key HIV outcomes, including appropriate ART prescription, viral suppression, performance of recommended health maintenance activities, and mortality were better among patients of HIV care providers with greater experience, defined as a larger HIV patient caseload and higher self-rated and objectively measured knowledge of HIV practices, but did not differ by provider profession or physician specialty [10–13, 22, 23]. However, this study demonstrates there are differences in whether needs for HIV case management and dental care are met among patients of various provider types, and these unmet needs could lead to suboptimal health outcomes and may be associated with lower quality life [1, 3, 8].

Compared with patients of ID physicians, a higher percentage of patients of NPs needed HIV case management, but fewer had an unmet need for that service, after accounting for availability of that service onsite at the facility where the providers practiced. This finding adds to existing literature examining ancillary services in which NPs were more likely than ID physicians to provide certain services essential for comprehensive HIV care such as ART adherence services, female reproductive counseling, sexual risk reduction services, and substance use risk reduction services [21]. Our study did not assess all these services, and we were not able to produce stable estimates of unmet needs for adherence support services due to small sample size for that particular service. However, since NPs are more likely to provide these comprehensive services, they might also be more likely to identify needs for other ancillary services, such as case management services, and make appropriate referrals when needed. Second, we found that fewer patients of non-ID physicians had unmet needs for dental care than patients of ID physicians, after accounting for the availability of onsite dental care at the facility where the physician practiced. Almost all non-ID physicians who treat PWH also provide primary care services to their patients, and fewer ID physicians report providing primary care services [21]. Attention to primary care can open more avenues during a visit to address patients’ health concerns beyond HIV, such as dental care. Clinical guidelines recommend taking an individualized approach to address each patient's specific needs and barriers to care to increase adherence to ART, including connecting patients with the necessary resources to reduce unmet needs [24]. To do this, providers should approach each patient with cultural humility and compassion to begin to understand, identify, and address the specific needs of patients from different cultural, social, and economic backgrounds. This may be helpful in addressing certain barriers to dental care. However, medical providers alone may not be able to fully mitigate the disparities in dental care, given many other barriers to dental care out of their control, such as costs and patients’ fear of or indifference to dental care [25].

Augmenting ID physician practices with other types of HIV care providers is one possible approach to better meet the need for HIV case management and dental care among people with HIV. Weiser et al [21] found that 80% of ID physicians report having sufficient time with patients to provide “necessary HIV information.” However, a focused approach to HIV care and treatment may tend to exclude discussions of, and support for, ancillary services that primary care physicians, NPs, and PAs are trained to address [26]. Strengthening ID practices with other provider types who are more likely to have primary care training and experience, and who are skilled in assessing and addressing the need for ancillary services, could lessen HIV provider disparities in meeting these needs. A 2014 study by Cheng et al found that 81% of insured patients at an academic HIV clinic supported colocation of a primary care provider at their HIV care facility, suggesting this type of structural intervention might have the potential to support HIV, primary care, and ancillary support needs [27].

Other strategies for ensuring patients of ID physicians receive needed HIV case management services and dental care include ongoing training, multidisciplinary case conferences, and use of decision support to nudge ID physicians to routinely assess their patients’ psychosocial needs and make appropriate referrals.

This analysis has limitations. Measures of ancillary service needs, sociodemographic characteristics, and some clinical characteristics were self-reported and subject to measurement bias. Data were cross-sectional, and thus, reported associations do not imply causality. Models measuring association of provider type with unmet needs for ancillary services were adjusted for patient and facility characteristics, but there may be residual confounding. Data were collected before and during the COVID-19 pandemic and might differ from data collected only during the pandemic. However, we have no reason to believe this would bias our estimates of differences in needs for ancillary services by provider type. Response rates were suboptimal, but results were adjusted for nonresponse and poststratified to known population totals by age, race/ethnicity, and sex from the NHSS using established, standard methodology. Even with suboptimal response rates, there is still value in results obtained from unbiased sampling methods [28]. The facility response rate was low. However, we used standard methods to impute facility characteristics for all patients analyzed.

## CONCLUSIONS

Patients of non-ID physicians, NPs, PAs, and mixed providers had greater need for many ancillary services than patients of ID physicians. After adjusting for differences in patient characteristics and the availability of onsite ancillary services at patients’ HIV care facilities, the proportion of needs that were unmet was generally comparable across groups. However, unmet need for HIV case management was lower among patients of NPs and unmet need for dental care was lower among patients of non-ID physicians compared with patients of ID physicians. Increasing the presence of NPs, PAs, and primary care physicians practicing with ID physicians could increase routine assessment of needs and referral for HIV case management and dental care and may improve HIV-related outcomes for PWH.

## Supplementary Data

Supplementary materials are available at *Open Forum Infectious Diseases* online. Consisting of data provided by the authors to benefit the reader, the posted materials are not copyedited and are the sole responsibility of the authors, so questions or comments should be addressed to the corresponding author.

## Supplementary Material

ofae284_Supplementary_Data

## Notes

***Acknowledgments*.** The authors thank the Medical Monitoring Project (MMP) participants and project area staff as well as members of the MMP advisory boards. The authors also acknowledge the contributions of the Clinical Outcomes Team and the Behavioral and Clinical Surveillance Branch at the Centers for Disease Control and Prevention (CDC).

***Author contributions*.** Study conception and design: C. T., S. D., D. J. R., and J. W. Data acquisition: S. D. and J. W. Data analysis: X. Y. and Y. T. Data interpretation: all authors. Drafting the manuscript: C. T., D. J. R., and J. W. Critical revisions and approval of the final manuscript: All authors.

***Patient consent*.** MMP data collection constitutes routine public health surveillance and was thus determined by the CDC to be nonresearch. This activity is conducted consistent with the applicable federal law and CDC policy. When required, participating jurisdictions obtained local institutional review board approval to collect data. All participants provided informed consent.

***Data availability*.** Data are not available publicly.

***Financial support*.** Funding for the Medical Montioring Project is provided by the Centers for Disease Control and Prevention. This research was supported in part by Infectious Diseases Society of America Foundation Grants for Emerging Researchers/Clinicians Mentorship Program (received by C. T.).
