# Supplementary material for: Unmet Needs for Ancillary Services by Provider Type Among People With Diagnosed Human Immunodeficiency Virus
Source: Open Forum Infect Dis. 2024 May 14;11(7):ofae284. doi: 10.1093/ofid/ofae284 (PMC11222969; doi:10.1093/ofid/ofae284)
Supplement: ofae284_Supplementary_Data [file ofae284_supplementary_data.docx]

| Supplementary Table 1. Unmet needs for ancillary care services by characteristics related to social determinants of health, behaviors, and clinical outcomes among adults diagnosed with HIV – United States 2019 (N=4100) | | | | | | | | | | | | | | | |
| --- | --- | --- | --- | --- | --- | --- | --- | --- | --- | --- | --- | --- | --- | --- | --- |
|  | **≥1 HIV support services unmet needs (N = 580)** | | | | | **≥1 non-HIV medical care services unmet needs (N = 1059)** | | | | | **≥1 subsistence services unmet needs (N = 717)** | | | | |
|  | **No.** | **wted row% (95% CI)** | **Unadjusted PD** ^a^ **(95% CI)** | **P** | **P overall** | **No.** | **wted row% (95% CI)** | **Unadjusted PD (95% CI)** | **P** | **P overall** | **No.** | **wted row% (95% CI)** | **Unadjusted PD (95% CI)** | **P** | **P overall** |
| **Total** | **580** | **15.7 (13.9-17.5)** | - | - | - | **1059** | **27.3 (24.9-29.7)** | - | - | - | **717** | **18.2 (16.0-20.3)** | - | - | - |
| **Age, in years** |  |  |  |  |  |  |  |  |  |  |  |  |  |  |  |
| 18-29 | 48 | 16.0 (11.5-20.5) | 1.7  (-2.7- 6.2) | 0.4452 | 0.0114 | 106 | 32.7 (26.4-38.9) | 9.1  (3.1- 15.1) | 0.003 | <.0001 | 80 | 22.4 (16.8-27.9) | 7.7  (2.4- 12.9) | 0.0041 | <.0001 |
| 30-39 | 118 | 20.4 (16.1-24.7) | 6.1  (2.0- 10.2) | 0.0034 | 0.0114 | 204 | 32.5 (28.7-36.3) | 8.9  (5.2- 12.7) | 0 | <.0001 | 139 | 23.2 (19.3-27.1) | 8.5  (4.5- 12.5) | 0 | <.0001 |
| 40-49 | 118 | 15.4 (12.5-18.2) | 1.1  ( -2.1- 4.3) | 0.5059 | 0.0114 | 230 | 30.6 (26.3-34.9) | 7.0  (2.3- 11.8) | 0.0035 | <.0001 | 173 | 21.2 (17.1-25.3) | 6.5  (2.9- 10.1) | 0.0004 | <.0001 |
| >=50 | 296 | 14.3 (12.3-16.3) | Reference | - | 0.0114 | 519 | 23.6 (20.8-26.3) | Reference | - | <.0001 | 325 | 14.7 (13.0-16.5) | Reference | - | <.0001 |
| **Gender** |  |  |  |  |  |  |  |  |  |  |  |  |  |  |  |
| Male | 405 | 15.4 (13.6-17.1) | Reference | - | 0.4204 | 758 | 27.4 (24.7-30.2) | Reference | - | 0.9631 | 467 | 16.6 (14.3-18.9) | Reference | - | 0.0004 |
| Female | 160 | 16.5 (13.6-19.5) | 1.2  (-1.7- 4.0) | 0.4217 | 0.4204 | 279 | 27.5 (24.0-31.0) | 0.1  (-3.8- 3.9) | 0.9631 | 0.9631 | 230 | 22.2 (19.3-25.1) | 5.6  (2.7- 8.5) | 0.0002 | 0.0004 |
| **Race/ethnicity** |  |  |  |  |  |  |  |  |  |  |  |  |  |  |  |
| White, non-Hispanic | 145 | 12.9 (10.2-15.6) | Reference | - | 0.0016 | 277 | 23.9 (20.0-27.9) | Reference | - | 0.0045 | 137 | 11.4 (9.1-13.7) | Reference | - | <.0001 |
| Black, non-Hispanic | 249 | 16.0 (12.6-19.3) | 3.1  ( -0.7- 6.9) | 0.1114 | 0.0016 | 467 | 29.5 (26.0-32.9) | 5.5  (0.7- 10.3) | 0.0238 | 0.0045 | 373 | 23.2 (19.5-26.8) | 11.8  (8.6- 14.9) | 0 | <.0001 |
| Hispanic or Latino | 133 | 15.6 (12.8-18.4) | 2.7  (-0.7- 6.2) | 0.1194 | 0.0016 | 227 | 24.6 (20.4-28.8) | 0.7  (-5.3- 6.6) | 0.8291 | 0.0045 | 135 | 15.2 (12.4-18.1) | 3.8  (0.9- 6.7) | 0.0105 | <.0001 |
| Other or Multiracial | 53 | 26.2 (20.0-32.5) | 13.3  (6.1- 20.6) | 0.0003 | 0.0016 | 88 | 38.0 (28.9-47.1) | 14.0  (5.8- 22.2) | 0.0008 | 0.0045 | 72 | 26.2 (19.5-33.0) | 14.8  (8.2- 21.5) | 0 | <.0001 |
| **Poverty threshold** ^b,c^ | | | | | |  |  |  |  |  |  |  |  |  |  |
| Above poverty level | 259 | 13.4 (11.7-15.2) | Reference | - | <.0001 | 509 | 25.8 (22.7-28.9) | Reference | - | 0.0954 | 260 | 12.3 (10.5-14.1) | Reference | - | <.0001 |
| At or below poverty level | 278 | 19.2 (16.4-22.0) | 5.8  (3.0- 8.5) | 0 | <.0001 | 442 | 28.9 (26.0-31.8) | 3.1  (-0.3- 6.5) | 0.0784 | 0.0954 | 374 | 25.2 (21.5-29.0) | 12.9  (9.7- 16.1) | 0 | <.0001 |
| **Educational attainment** | | | | | | | |  |  |  |  |  |  |  |  |
| <High school | 94 | 15.1 (12.0-18.2) | -1.1  (-4.4- 2.2) | 0.5154 | 0.6515 | 193 | 29.3 (25.7-33.0) | 3.4  (-0.5- 7.3) | 0.0862 | 0.098 | 158 | 23.3 (18.5-28.1) | 7.7  (3.5- 12.0) | 0.0004 | <.0001 |
| High school diploma or equivalent | 151 | 15.0 (11.9-18.1) | -1.2  (-4.3- 1.9) | 0.4431 | 0.6515 | 312 | 29.2 (25.6-32.7) | 3.3  (-0.4- 7.0) | 0.082 | 0.098 | 221 | 20.6 (17.9-23.3) | 5.1  (2.1- 8.1) | 0.001 | <.0001 |
| >High school | 335 | 16.2 (14.4-18.0) | Reference | - | 0.6515 | 554 | 25.9 (23.0-28.8) | Reference | - | 0.098 | 338 | 15.6 (13.3-17.8) | Reference | - | <.0001 |
| **Health insurance coverage** ^d^ | | | | | | | | |  |  |  |  |  |  |  |
| Any private | 162 | 12.2 (10.1-14.2) | Reference | - | <.0001 | 281 | 22.1 (19.0-25.2) | Reference | - | 0.0004 | 127 | 9.2  (7.3-11.2) | Reference | - | <.0001 |
| Public only | 351 | 17.2 (14.7-19.8) | 5.1  (2.3- 7.8) | 0.0003 | <.0001 | 634 | 28.9 (25.6-32.3) | 6.8  (2.7- 11.0) | 0.0012 | 0.0004 | 495 | 22.6 (19.5-25.8) | 13.4  (10.3- 16.5) | 0 | <.0001 |
| Ryan White/ADAP only | 34 | 9.9  (6.7-13.2) | -2.2  (-6.4- 1.9) | 0.2943 | <.0001 | 113 | 31.8 (24.0-39.6) | 9.7  (2.9- 16.6) | 0.0055 | 0.0004 | 71 | 21.5 (15.6-27.4) | 12.3  (6.4- 18.1) | 0 | <.0001 |
| No Coverage/Uninsured | 27 | 60.5 (43.3-77.8) ^i^ | 48.4  (31.2- 65.5) | 0 | <.0001 | 19 | 48.6 (32.0-65.1) ^i^ | 26.5  (9.1- 43.8) | 0.0028 | 0.0004 | 13 | 25.1 (13.1-37.1) | 15.9  (3.6- 28.1) | 0.0111 | <.0001 |
| **Homelessness in the past 12 months** | | | | | |  |  |  |  |  |  |  |  |  |  |
| Yes | 170 | 23.3 (20.1-26.6) | 9.5  (6.0- 13.0) | 0 | <.0001 | 296 | 39.5 (35.1-43.9) | 15.2  (11.2- 19.2) | 0 | <.0001 | 276 | 37.0 (32.1-42.0) | 23.6  (19.1- 28.0) | 0 | <.0001 |
| No | 410 | 13.8 (12.0-15.7) | Reference | - | <.0001 | 763 | 24.3 (22.1-26.5) | Reference | - | <.0001 | 441 | 13.5 (11.8-15.2) | Reference | - | <.0001 |
| **Incarceration in the past 12 months** | | | | | | | |  |  |  |  |  |  |  |  |
| Yes | 44 | 31.5 (22.2-40.8) | 16.4  (7.5- 25.3) | 0.0003 | <.0001 | 51 | 35.4 (25.2-45.6) | 8.4  (-1.2- 18.0) | 0.0853 | 0.059 | 37 | 25.0 (16.9-33.1) | 7.1  (-0.4- 14.6) | 0.0639 | 0.0336 |
| No | 535 | 15.0 (13.4-16.7) | Reference | - | <.0001 | 1007 | 27.0 (24.7-29.3) | Reference | - | 0.059 | 680 | 17.9 (15.8-20.0) | Reference | - | 0.0336 |
| **Drug use in the past 12 months** | | | | | | | | |  |  |  |  |  |  |  |
| Yes | 240 | 20.4 (18.1-22.7) | 7.1  (4.4- 9.7) | 0 | <.0001 | 444 | 35.9 (32.6-39.2) | 12.7  (8.7- 16.6) | 0 | <.0001 | 292 | 23.6 (20.6-26.7) | 8.2  (4.4- 12.0) | 0 | <.0001 |
| No | 334 | 13.3 (11.4-15.3) | Reference | - | <.0001 | 608 | 23.2 (20.6-25.9) | Reference | - | <.0001 | 420 | 15.4 (13.0-17.9) | Reference | - | <.0001 |
| **Injection drug use in the past 12 months** | | | |  |  |  |  |  |  |  |  |  |  |  |  |
| Yes | 30 | 31.4 (18.3-44.5) | 16.1  (3.5- 28.8) | 0.0124 | 0.001 | 54 | 53.3 (39.5-67.1) | 26.6  (13.6- 39.6) | 0.0001 | <.0001 | 32 | 28.0 (18.7-37.3) | 10.1  (0.9- 19.3) | 0.0314 | 0.0142 |
| No | 547 | 15.3 (13.7-16.9) | Reference | - | 0.001 | 1004 | 26.7 (24.4-29.0) | Reference | - | <.0001 | 682 | 17.9 (15.8-20.0) | Reference | - | 0.0142 |
| **Binge drinking ^e^** |  |  |  |  |  |  |  |  |  |  |  |  |  |  |  |
| Yes | 93 | 18.3 (14.0-22.5) | 3.0  (-1.1- 7.0) | 0.1482 | 0.1205 | 196 | 33.9 (28.7-39.1) | 7.7  (2.8- 12.7) | 0.0023 | 0.0011 | 116 | 20.6 (15.7-25.6) | 3.0  (-1.6- 7.6) | 0.2052 | 0.173 |
| No | 483 | 15.3 (13.6-17.0) | Reference | - | 0.1205 | 857 | 26.2 (23.8-28.5) | Reference | - | 0.0011 | 595 | 17.7 (15.7-19.7) | Reference | - | 0.173 |
| **Major or other depression symptoms in the past 2 weeks ^f^** | | | | | | | | | | | |  |  |  |  |
| Yes | 189 | 31.4 (27.1-35.7) | 18.9  (14.9- 22.9) | 0 | <.0001 | 288 | 45.8 (41.7-49.9) | 22.0  (18.0- 26.1) | 0 | <.0001 | 216 | 33.2 (28.8-37.6) | 18.1  (14.1- 22.1) | 0 | <.0001 |
| No | 383 | 12.5 (11.0-14.0) | Reference | - | <.0001 | 760 | 23.8 (21.6-26.0) | Reference | - | <.0001 | 491 | 15.1 (13.4-16.8) | Reference | - | <.0001 |
| **Moderate or severe generalized anxiety symptoms in the past 2 weeks ^g^** | | | | | | | | | | |  |  |  |  |  |
| Yes | 177 | 30.3 (26.2-34.3) | 17.3  (13.1- 21.5) | 0 | <.0001 | 288 | 46.0 (41.3-50.8) | 22.2  (17.6- 26.9) | 0 | <.0001 | 204 | 30.2 (26.3-34.0) | 14.3  (9.9- 18.8) | 0 | <.0001 |
| No | 400 | 13.0 (11.5-14.5) | Reference | - | <.0001 | 766 | 23.8 (21.8-25.8) | Reference | - | <.0001 | 510 | 15.8 (13.7-18.0) | Reference | - | <.0001 |
| **Attended RWHAP -funded facility ^i^** | | | | | | | |  |  |  |  |  |  |  |  |
| Yes | 363 | 15.5 (13.5-17.5) | Reference | - | 0.9072 | 700 | 27.8 (24.9-30.7) | Reference | - | 0.4881 | 502 | 19.5 (16.8-22.1) | Reference | - | 0.0049 |
| No | 175 | 15.7 (12.9-18.6) | 0.2  (-3.4- 3.9) | 0.9078 | 0.9072 | 285 | 26.3 (23.1-29.5) | -1.5  (-5.4- 2.5) | 0.4714 | 0.4881 | 165 | 14.8 (12.4-17.3) | -4.7  ( -7.2- -2.1) | 0.0004 | 0.0049 |

Abbreviations: No, sample size; wted, weighted; CI, confidence interval; PD, prevalence difference; ID, infectious disease; RWHAP, Ryan White HIV/AIDS Program

^a^ Prevalence difference of categories compared with a reference group.

^b^ Poverty guideline as defined by the Department of Health and Human Services; https://aspe.hhs.gov/topics/poverty-economic-mobility/poverty-guidelines

^c^ During the last 12 months

^d^ Participants were able to select more than once response for health insurance/medication coverage.

^e^ Binge drinking is defined as 5 or more alcohol drinks in one sitting for men, 4 or more alcohol drinks in one sitting for women.

^f^ Depression symptoms based on PHQ-9

^g^ Anxiety symptoms based on the GAD-7

^h^ Any RWHAP funding (A, B, C, D, or F)

^i^ Coefficient of variation is ≥0.3, absolute confidence interval (CI) width is ≥0.30, or absolute CI width is between 0.05 and 0.30 and relative CI width is >130%. Estimate should be interpreted with caution.
